# The association between mechanical ventilator compatible bed occupancy and mortality risk in intensive care patients with COVID-19: a national retrospective cohort study

**DOI:** 10.1186/s12916-021-02096-0

**Published:** 2021-08-30

**Authors:** Harrison Wilde, Thomas Mellan, Iwona Hawryluk, John M. Dennis, Spiros Denaxas, Christina Pagel, Andrew Duncan, Samir Bhatt, Seth Flaxman, Bilal A. Mateen, Sebastian J. Vollmer

**Affiliations:** 1 Department of Statistics, University of Warwick, Coventry, CV4 7AL UK; 2 MRC Centre for Global Infectious Disease Analysis, Abdul Latif Jameel Institute for Disease and Emergency Analytics (J-IDEA), Imperial College London, London, UK; 3 Institute of Biomedical & Clinical Science, RILD Building, Royal Devon & Exeter Hospital, University of Exeter Medical School, Barrack Road, Exeter, EX2 5DW UK; 4 The Alan Turing Institute, British Library, 96 Euston Road, London, NW1 2DB UK; 5 Institute of Health Informatics, University College London, 222 Euston Rd, London, London NW1 2DA UK; 6 Health Data Research UK, Gibbs Building, 215 Euston Road, London, NW1 2BE UK; 7 Clinical Operational Research Unit, University College London, 222 Euston Rd, London, London NW1 2DA UK; 8 Department of Mathematics, Imperial College, London, London, UK; 9 The Wellcome Trust, Gibbs Building, 215 Euston Road, London, NW1 2BE UK

**Keywords:** Critical care, Hospital mortality, Public health surveillance, Quality of healthcare, Coronavirus infection

## Abstract

**Background:**

The literature paints a complex picture of the association between mortality risk and ICU strain.

In this study, we sought to determine if there is an association between mortality risk in intensive care units (ICU) and occupancy of beds compatible with mechanical ventilation, as a proxy for strain.

**Methods:**

A national retrospective observational cohort study of 89 English hospital trusts (i.e. groups of hospitals functioning as single operational units). Seven thousand one hundred thirty-three adults admitted to an ICU in England between 2 April and 1 December, 2020 (inclusive), with presumed or confirmed COVID-19, for whom data was submitted to the national surveillance programme and met study inclusion criteria. A Bayesian hierarchical approach was used to model the association between hospital trust level (mechanical ventilation compatible), bed occupancy, and in-hospital all-cause mortality. Results were adjusted for unit characteristics (pre-pandemic size), individual patient-level demographic characteristics (age, sex, ethnicity, deprivation index, time-to-ICU admission), and recorded chronic comorbidities (obesity, diabetes, respiratory disease, liver disease, heart disease, hypertension, immunosuppression, neurological disease, renal disease).

**Results:**

One hundred thirty-five thousand six hundred patient days were observed, with a mortality rate of 19.4 per 1000 patient days. Adjusting for patient-level factors, mortality was higher for admissions during periods of high occupancy (> 85% occupancy versus the baseline of 45 to 85%) [OR 1.23 (95% posterior credible interval (PCI): 1.08 to 1.39)]. In contrast, mortality was decreased for admissions during periods of low occupancy (< 45% relative to the baseline) [OR 0.83 (95% PCI 0.75 to 0.94)].

**Conclusion:**

Increasing occupancy of beds compatible with mechanical ventilation, a proxy for operational strain, is associated with a higher mortality risk for individuals admitted to ICU. Further research is required to establish if this is a causal relationship or whether it reflects strain on other operational factors such as staff. If causal, the result highlights the importance of strategies to keep ICU occupancy low to mitigate the impact of this type of resource saturation.

**Supplementary Information:**

The online version contains supplementary material available at 10.1186/s12916-021-02096-0.

## Background

From the first reports of a novel coronavirus (SARS-CoV-2) in late 2019, to date, global mortality associated with the resultant disease (COVID-19) has exceeded 1.7 million people [1]. The virulence of the pathogen has prompted persistent concern about the ability of health services around the world to effectively care for the vast numbers of people affected [2]. These concerns are most relevant in the context of scarce resources (e.g. mechanical ventilation) required by patients in need of high-acuity support, which is relatively common in patients with COVID-19 [3]. Notably, even with the introduction of non-pharmacological interventions such as stay-at-home orders, almost a third of all hospitals in England reached 100% occupancy of their ‘surge’ mechanical ventilation capacity (i.e. including the additional beds that were created through the re-allocation of resources) during the first wave of the pandemic [4]. The second wave in England appears to have been worse than the first, with 40% more people in hospital, many hospitals overwhelmed and staff wellbeing suffering due to the prolonged onslaught [5]. What remains unclear is whether and to what extent operating at these extremes of critical care occupancy impacted patient outcomes.

Availability of certain resources, such as bed occupancy/availability, is well-established proxies for operational strain [6]. Prior to COVID-19, the literature painted a complex picture of the association between morbidity and mortality and ICU strain [7]. Some studies suggest that higher strain in intensive care was associated with increased morbidity and mortality risk [8–10], whereas others observed no association at all [11, 12]. Data from the natural experiment caused by the COVID-19 pandemic is accumulating; however, it is similarly inconsistent. A recent study from Belgium reported 42% higher mortality during periods of ICU surge capacity deployment, although in the analysis utilisation of surge capacity was only evaluated as a binary variable [13]. Similarly, a study based on the US Veterans Affairs database evaluating quartile-based bins for occupancy found a 94% increased risk of mortality when occupancy exceeded 75% [14]. The aforementioned observations both directly contradict earlier results from smaller studies in Australia and Wales, where no association between ICU occupancy and mortality was identified [15, 16].

A better understanding of how operating under such extreme circumstances affects outcomes is crucial for two reasons: firstly, to allow hospitals to adapt practice to improve outcomes, and secondly, to provide policy makers with more accurate information about the potential consequences of allowing health services to be overwhelmed. As such, in this study, we sought to evaluate the extent to which mortality risk in intensive care units (ICUs) during the COVID-19 pandemic in England could be explained by differences in occupancy.

## Methods

### Data sources

Data on all intensive care unit (ICU) admissions across England were extracted from the COVID-19 Hospitalisation in England Surveillance System (CHESS) [17]. Information on occupancy rates were extracted from the daily situation reports (i.e. ‘SitRep’) [4]. Both datasets are mandatory regulatory submissions for all National Health Service (NHS) acute care providers in England, and further details about them can be found in additional file 1: eMethods.

### Study population

All ICU admissions reported to CHESS between 2 April and 1 December (see additional file 1: eMethods for details on date selection), with presumed/confirmed COVID-19 (100% tested positive during their admission; see additional file 1: eMethods for details on diagnosis), aged 18–99, non-pregnant, and with valid admission and occupancy data, were eligible for inclusion (additional file 1: Fig. S1).

### Recorded clinical features

#### Patient-level data

Information extracted from CHESS about each patient comprised: administrative features (admitting trust, admission date, and first segment of postcode—used to identify the relevant indices of multiple deprivation for the corresponding areas in England)), demographic characteristics (age, sex, ethnicity), recorded comorbidities (obesity, diabetes, asthma, other chronic respiratory disease, chronic heart disease, hypertension, immunosuppression due to disease or treatment, chronic neurological disease, chronic renal disease, chronic liver disease), and a binary indicator for use of mechanical ventilation. Ethnicity was coded as white, Asian (Subcontinent and other), black, mixed, and other; comorbidities were recorded as binary indicator variables, with missing entries assumed to reflect the absence of a comorbidity. The appropriateness of this assumption in CHESS has been previously explored [18].

#### Occupancy data

Trusts are groups of geographically co-located hospitals that function as a single organisational unit within the UK’s national healthcare system. Information extracted from SitRep about each trust comprised: pre-pandemic (January–March 2020) number of beds compatible with mechanical ventilation, the proportion of beds compatible with mechanical ventilation occupied on each day of the study period, and each trust’s geographical region [4]. Linkage was carried out based on the trust that an individual was admitted to and the date of ICU admission in CHESS; patients in CHESS were matched via their admission date to the relevant occupancy information from the corresponding date in SitRep.

#### Outcome

The primary outcome was in-hospital all-cause mortality; patients were followed up until death, discharge, transfer, or a minimum of 90 days from admission. Discharge and transfer were both treated as suggesting that the patient survived.

### Statistical analysis

Descriptive summaries were generated as median and interquartile ranges for continuous variables and frequency and percentage incidence for categorical factors. Exploratory analyses included: the relationship of the COVID-19 epidemic curve to bed occupancy at a national level (additional file 1: Fig. S2); the distribution of missingness amongst patients and trusts (additional file 1: Fig. S3); variation in age and comorbidity burden over the first wave (additional file 1: Fig. S4); the impact of modelling continuous variables either linearly, through the use of threshold functions, or via (standard cubic) splines/smooths (additional file 1: eMethods).

A Bayesian hierarchical approach was used to model the association between the trust, group and patient-level factors and mortality risk. Specifically, a generalised additive mixed model was utilised, with intercept and slope coefficients for population and group level effects, and a Bernoulli likelihood with logit function to link to mortality outcome. Coefficients were inferred by Markov chain Monte Carlo sampling, using Stan (CmdStan V2.25.0), with a model specified using BRMS (V2.14.4) in R (V4.0.3) [19–21]. Further information on the Bayesian prior specification and modelling methods are reported in additional file 1: eMethods. The rationale for the different thresholds utilised for bed occupancy is also detailed in additional file 1: eMethods; in short, the 85% occupancy threshold utilised is based on guidance from the Royal College of Emergency Medicine [22], whereas the 45% (low occupancy threshold) was derived from the exploratory analysis.

As secondary analyses, two potential interactions were assessed: (1) baseline trust size and occupancy and (2) patient age and occupancy (results not reported due to insignificance). We also assessed the association of occupancy on the recorded outcome date with mortality and occupancy expressed in terms of pre-pandemic ICU size. Several sensitivity analyses were carried out (see additional file 1: eMethods for justifications): (1) filtering for different degrees of missingness of patient-level comorbidity data at trust-level; (2) including all individuals still on the unit as of 2 March (and assuming they all survive or die; *n* = 250); (3) including all individuals with a known outcome but no date whom were excluded in the data cleaning process (*n* = 10); (4) a random effect for all patient post 16 June 2020 (i.e. the date of the Recovery Trials press-release regarding the effectiveness of dexamethasone); (5) adjusting for week of admission; (6) adjusting for trust and region as random effects; (7) additional patient-level factors: time from hospital admission to ICU admission, chronic liver disease and obesity; (8) the residual bed availability in the sustainability and transformation partnership (STPs) to which each trust belongs—STPs are collections of geographically co-located hospital trusts that are meant to operate collaboratively to support one another, and include ambulance providers and community services so as to allow end-to-end integration of care; (9) the weighted average of the indices of multiple deprivation (IMD) corresponding to each patient’s given postcode district (i.e. the first half of their postcode), as a proxy for socio-economic status; and (10) separated into the different waves of the pandemic in the UK (i.e. 1st, inter-wave/summer, and 2nd).

## Results

Seven thousand one hundred thirty-three individuals were included in this study following application of the inclusion/exclusion criteria (see additional file 1: Fig. S1), of whom 2637 (37.0%) died. In total, 135,600 (median 12 days per patient; IQR 6–25) patient days were observed, equating to a mortality rate of 19.4 per 1000 patient days. A full summary of the recorded patient-level characteristics is reported in Table 1. Occupancy of beds compatible with mechanical ventilation over the study observation period is illustrated in additional file 1: Fig. S5, expanding on the results presented elsewhere which are restricted to the first wave [4].

**Table 1:** Characteristics of the study cohort stratified by occupancy on the day of admission

	Occupancy			
	0–45%	45–85%	85–100%	Overall
	(*n* = 1602)	(*n* = 4442)	(*n* = 1089)	(*n* = 7133)
**Age in years**				
Median [IQR]	60 [51, 69]	60 [51, 69]	60 [52, 68]	60 [51, 69]
**Time in days to ICU from hospital admission**				
Median [IQR]	1 [0, 4]	1 [0, 3]	1 [0, 3]	1 [0, 3]
**Age group**				
18–24	13 (0.8)	45 (1.0)	11 (1.0)	69 (1.0)
25–34	71 (4.4)	148 (3.3)	37 (3.4)	256 (3.6)
35–44	134 (8.4)	365 (8.2)	81 (7.4)	580 (8.1)
45–54	331 (20.7)	894 (20.1)	227 (20.8)	1452 (20.4)
55–64	443 (27.7)	1381 (31.1)	340 (31.2)	2164 (30.3)
65–74	393 (24.5)	1084 (24.4)	293 (26.9)	1770 (24.8)
75–84	193 (12.0)	452 (10.2)	87 (8.0)	732 (10.3)
85+	24 (1.5)	73 (1.6)	13 (1.2)	110 (1.5)
**Sex**				
Female	545 (34.0)	1402 (31.6)	320 (29.4)	2267 (31.8)
Male	1057 (66.0)	3040 (68.4)	769 (70.6)	4866 (68.2)
**Ethnicity**				
White	1088 (67.9)	2628 (59.2)	506 (46.5)	4222 (59.2)
Asian subcontinent	120 (7.5)	466 (10.5)	137 (12.6)	723 (10.1)
Asian (other)	75 (4.7)	260 (5.9)	85 (7.8)	420 (5.9)
Black	61 (3.8)	286 (6.4)	113 (10.4)	460 (6.4)
Mixed	14 (0.9)	77 (1.7)	37 (3.4)	128 (1.8)
Other	76 (4.7)	264 (5.9)	69 (6.3)	409 (5.7)
Missing	168 (10.5)	461 (10.4)	142 (13.0)	771 (10.8)
**Obesity**				
Obese	652 (40.7)	1766 (39.8)	454 (41.7)	2872 (40.3)
Non-obese	520 (32.5)	1665 (37.5)	312 (28.7)	2497 (35.0)
Missing	430 (26.8)	1011 (22.8)	323 (29.7)	1764 (24.7)
**Comorbidity**				
Diabetes	366 (22.8)	1184 (26.7)	304 (27.9)	1854 (26.0)
Chronic respiratory disease(s)	378 (23.6)	902 (20.3)	226 (20.8)	1506 (21.1)
Chronic heart disease	194 (12.1)	548 (12.3)	103 (9.5)	845 (11.8)
Chronic renal disease	124 (7.7)	384 (8.6)	78 (7.2)	586 (8.2)
Chronic neurological disease	105 (6.6)	216 (4.9)	37 (3.4)	358 (5.0)
Chronic liver disease	81 (5.1)	91 (2.0)	18 (1.7)	190 (2.7)
Immunosuppressive disease	61 (3.8)	146 (3.3)	20 (1.8)	227 (3.2)
Hypertension	534 (33.3)	1576 (35.5)	328 (30.1)	2438 (34.2)
**Invasive mechanical ventilation**				
Proportion ventilated	699 (43.6)	2296 (51.7)	666 (61.2)	3661 (51.3)
**Mortality**				
Crude (unadjusted, absolute)	522 (32.6)	1654 (37.2)	461 (42.3)	2637 (37.0)

Continuous covariates are presented with their median and interquartile range, whilst categorical covariates are presented with their frequency and within column percentage prevalence

### Mechanical ventilator occupancy rate on the day of admission is associated with mortality risk

**Fig. 1:**
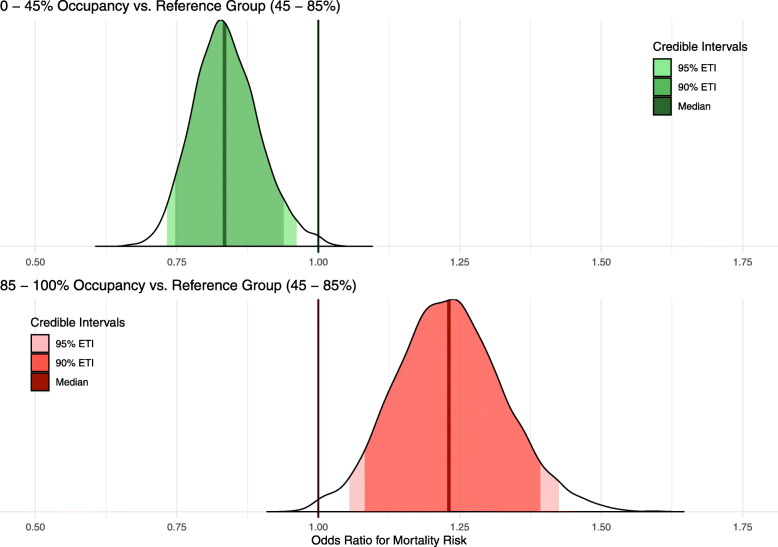
The adjusted odds ratios for the risk of mortality based on different ICU bed occupancy rates (treated as a three-level categorical variable). The full posterior distribution of the odds ratio (OR) for mortality given low occupancy 0–45% (top; green), and high occupancy 85–100% (bottom; red) are presented. The PCIs presented are equally tailed credibility intervals for the posterior odds ratio distributions. The outer (less saturated) interval is the 95% PCI, and the inner (more saturated) interval shows the 90% PCI. The reference category is 45–85% occupancy. Exact values are tabulated

**Fig. 2:**
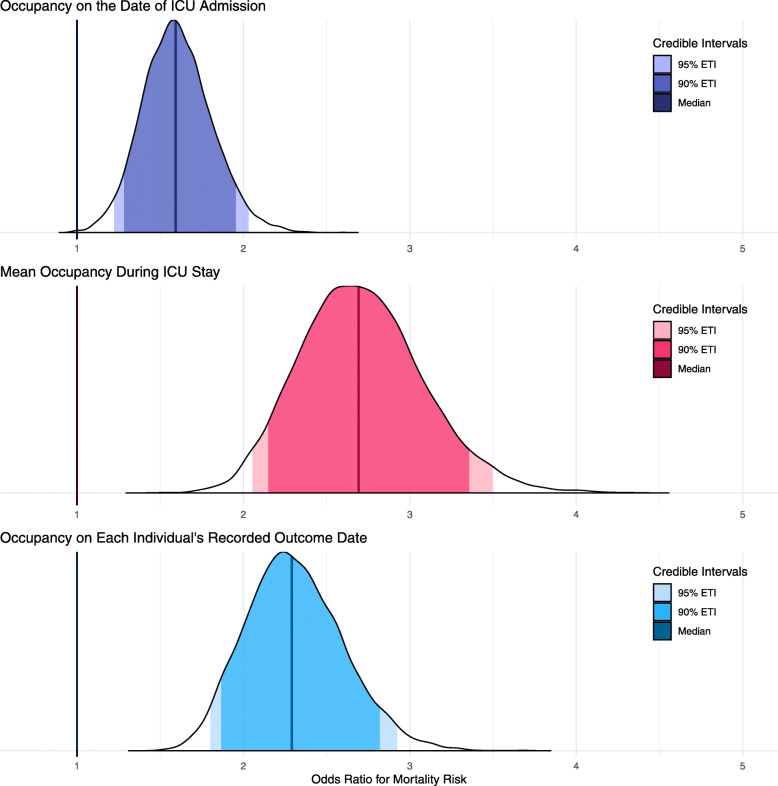
The adjusted odds ratios for the risk of mortality based on ICU bed occupancy (treated as a linear continuous variable) on the day of admission (top) and each individual’s recorded outcome date (bottom). The full posterior distribution of the odds ratio (OR) for mortality given occupancy on the date of ICU admission (top; purple), mean occupancy during ICU stay (middle: pink), and occupancy on the date of each individual’s recorded outcome (bottom; blue) are presented. The PCIs presented are equally tailed credibility intervals for the posterior odds ratio distributions. Occupancy was specified without multiplying out by 100 (i.e. 20% = 0.20); therefore, the odds ratio is for a step from 0% to 100% (i.e. 0.0 to 1.0). Exact values are tabulated

For high occupancy rates (85–100% of total beds, including surge capacity), the unadjusted odds ratio (OR) for mortality based on the mechanical ventilator occupancy rate on the day of admission was 1.22 (95% posterior credibility interval (PCI) 1.06–1.40), relative to the baseline of 45–85%. For low occupancy rates (0–45%), the unadjusted odds ratio was 0.82 (95% PCI 0.73–0.93), relative to the baseline of 45–85%. Following adjustment for patient-level factors (age, sex, ethnicity, and comorbidities), the OR was 1.23 (95% PCI 1.05–1.43) for high occupancy rates and 0.83 (95% PCI 0.73–0.96) for low occupancy rates. Figure 1 illustrates the posterior probabilities for the fully adjusted ORs (see additional file 1: Fig. S6 & S7 for other model coefficients). To aid interpretation, the difference in risk for a 70-year-old man with no comorbidities being admitted during a period of high versus low occupancy is equivalent to the risk of them being over a decade older (see full relationship in Fig. 2). Sensitivity analyses as detailed in additional file 1: Table S1 were all concordant with the primary analysis, and the results do not appear to be sensitive to the choice of prior as illustrated in additional file 1: Table S2.

### Mortality risk increases linearly with admission date specific occupancy and average exposure over the full length of stay

**Fig. 3:**
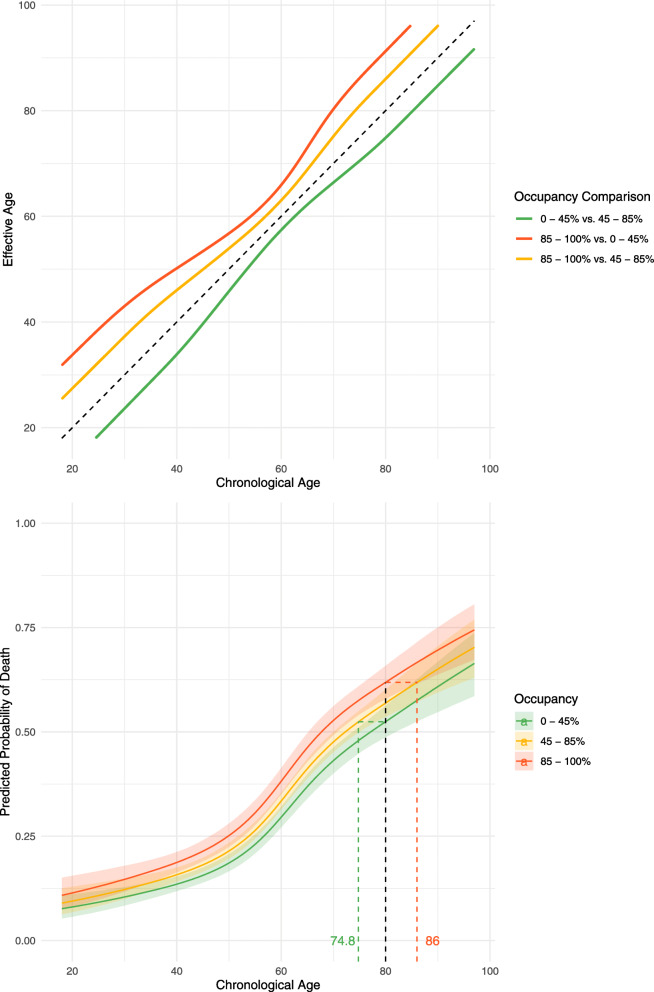
The increase in mortality risk associated with admission to intensive care during periods of different occupancy rates, expressed in terms of the equivalent increase in years of age. (Left) The predicted mortality curves arising from predictions made by the primary model across a range of age values for a white male patient is shown alongside 95% credible intervals in a ribbon either side of the median line. The black dotted line intersects all three curves; the 0–45% and 85–100% occupancy curve *y* value probabilities can then be used to solve back onto the reference curve to determine effective ages of equal risk to the chosen age under reference 45–85% occupancy, shown by the corresponding green and red dotted lines respectively [23]. (Right) The plot illustrates the number of years of additional age that ICU admission on a day with each different mechanical ventilation bed occupancy rate equates to. For example, an individual with a chronological age of 40 has an effective age of 31 in a low occupancy setting (green) and 45 in a high occupancy setting (orange). Both of the aforementioned comparisons are relative to the baseline occupancy of 45–85%). A comparison of the difference in risk between being admitted to the highest occupancy range relative to the lowest is shown in (red) and for a 40-year-old is equivalent to an increase in their age by 11 years

The fully adjusted OR for mortality (Fig. 3), using occupancy on the day of admission coded as a continuous linear variable ranging from 0 to 1 (i.e. specified without multiplying out by 100 (e.g. 20% = 0.20), therefore the resulting odds ratio is for a step from 0% to 100%), was 1.59 (95% PCI 1.22–2.03). Furthermore, using the occupancy from each individuals’ outcome date identified an even larger association (full model specification in additional file 1: Table S3), OR 2.29 (95% PCI 1.80–2.92). Finally, the fully adjusted OR for the mean occupancy over a patient’s total length of stay in ICU was 2.69 (95% PCI 2.05–3.50); see additional file 1: Table S4.

### Mortality risk is evident regardless of comparison to surge or baseline capacity as the reference

Using the alternative definition of occupancy, relative to baseline (i.e. pre-pandemic) capacity instead of the real-time (surge) capacity on the day of admission illustrates that mortality risk given a linear continuous factor was 1.31 (95% PCI 1.16–1.48; additional file 1: Table S5). In other words, every 100% increase in occupancy above baseline capacity was associated with a 31% increase in risk of mortality.

### Larger ICUs experience exaggerated impacts of extremes of mechanical ventilator occupancy rates

Pre-pandemic number of beds did not substantially alter the OR of occupancy as a sensitivity analysis (additional file 1: Table S1). Moreover, for every additional 25 beds compatible with mechanical ventilation (pre-pandemic), there was no observed (significant at the 5% threshold) decrease in mortality (additional file 1: Table S6). However, introduction of an interaction term between pre-pandemic size and real-time occupancy identified that larger ICUs experience exaggerated impacts of extremes of mechanical ventilator occupancy rates (additional file 1: Fig. S8).

## Discussion

The results of this study, including the individual-level experience of over 7000 patients admitted to intensive care units across England, suggest that survival rates for patients with COVID-19 in intensive care settings appears to deteriorate as the occupancy of (surge capacity) beds compatible with mechanical ventilation (a proxy for operational pressure [6]), increases. Moreover, this risk does not occur above a specific threshold, but rather appears linear, whereby going from 0% occupancy to 100% occupancy increases the odds of mortality by 59% (after adjusting for relevant individual-level factors). Furthermore, and expectedly, greater exposure to periods of high occupancy over an individual’s total length of stay is also associated with an increased risk of mortality. This lends credibility (via a potential dose-response relationship) to the argument that this is a causal relationship that is being observed; however, we cannot make a definite claim based on our results. An additional piece of context for interpreting these results is that the relevant authorities in the UK made clear that no explicit triaging unique to the pandemic took place [24], meaning that everyone whom would have previously been deemed suitable for ICU treatment was treated similarly to prior to the pandemic.

The remaining result for which the interpretation is not immediately apparent is that the risk of mortality based on occupancy on the date of recorded outcome is also high; OR 2.29 (95% posterior credible interval 1.80–2.92). There are several plausible explanations, but for the sake of brevity, we will mention just two. The first is based on the same principle as the other results, i.e. as occupancy increases the availability of staff and resources decreases which can indirect contribute to an individual’s mortality risk, and/or there may be a direct effect due to rationing of care or treatment limiting orders. Alternatively, this result could also reflect a specific type of observation bias wherein patients who are likely to survive are kept in ICU and in hospital longer [25], thus resulting in them being eventually discharged during periods of lower occupancy. In both instances further research is needed to clarify the underlying causal mechanism which is driving these observations.

### In context of the literature

Our observations are consistent with the aforementioned Belgian and US VA study [13, 14], except that they extend the results to suggest a linear association rather than stepped increase at specific thresholds. Although previous COVID-19 specific studies report an association between larger ICUs and lower COVID-19 mortality [26], our results are equivocal. However, we did observe an interaction with (pre-pandemic) unit size, whereby larger ICUs experience more exaggerated impacts from both higher and lower (surge) occupancy rates, although it is unclear from our data what is driving this heterogeneity. Finally, these results might partially explain the decreased mortality rate seen in the latter half of the first wave in the UK [27], where occupancy rates were much lower than at the peak [4]. Importantly, the average number of co-morbidities per patient appears to rise when occupancy decreases (additional file 1: Fig. S4), suggesting if there was implicit/soft triaging it would not have been in a manner that would explain the observed effect (i.e. more co-morbidities are generally thought to be associated with a worse outcome). However, more research is necessary to definitively exclude this potential explanation for the results.

### Strengths and limitations

The strengths of this study are the national cohort of patient-level data with extensive capture of admissions [25], coupled with a rigorous modelling method (additional file 1: eMethods). However, there are also several limitations. For example, we lack 30-day outcome data for discharged and transferred individuals and thus were forced to model under a naïve assumption that these individuals survived, which may have impacted our estimates of the risk of mortality. Below is a more detailed discussion on three key issues that should be accounted for when interpreting the associations reported in this study:

#### Availability of physiological and clinical data

The lack of physiological data and minimal clinical intervention data available in the CHESS dataset limits our ability to adjust for individual-level differences in severity and treatment. Thus, we cannot exclude that the observed associations are a result of unmeasured confounding due to differences in severity on admission or variation in natural history due to the evolving treatment strategies that have been used over the course of the pandemic. At present, there is no single research environment that links primary care, secondary care, administrative mortality records, surveillance datasets (e.g. CHESS), routine COVID testing (e.g. Public Health England’s testing data), and national audit data such as that collected by the UK Intensive Care National Audit and Research Centre (ICNARC) which includes some of the necessary information at the patient-level. Notably, there are on-going attempts to link the aforementioned datasets, e.g. [28]. However, this exercise is still not complete, and at the time of writing this, the (linked) ICNARC dataset is not available. In essence, a comprehensive analysis including physiological and treatment data alongside all the other ancillary datasets is not possible. To our knowledge, the UK consortium is the first attempt to make such a national dataset and thus replication of our analysis with these data once available is a critical future task.

Although direct analysis to address the above issues is not possible for the reasons outlined previously, it is still worth noting that several studies from the UK suggest that any potential differences in severity on admission are unlikely to explain the observed association between occupancy and mortality. For example, a study using linked CHESS data from the first wave of the pandemic did not find between centre variation in severity scores (e.g. mean APACHE-II) to be associated with mortality risk [29]. Moreover, the UK national audit (ICNARC) did not identify any significant temporal variation in mean severity scores of patients admitted to ICU over the course of this study [30]; both results in combination suggest that unmeasured confounding due to differences in severity of admission are unlikely to fully explain the observed association.

#### Incomplete characterisation of operational strain

Characterisation of operational strain as a function of surge occupancy likely fails to fully reflect the complexity of using non-specialist staff and other resource allocation issues present when ‘creating’ new ICU beds, which could be the underlying causal explanation for the increased mortality risk observed during periods of high occupancy. Notably, previous research has identified a detrimental impact of insufficient (adequately trained) nursing and medical consultant staff on patient mortality risk in intensive care units [31], and staff absence rates were raised 3-fold from the baseline of 4% at the peak of the first wave of the pandemic [32]. As such, it is not just functional strain due to the creation of new beds, but also absence that could underlie a staff-specific explanation for the observed association. Again, this is a key area for future research to explore, especially with mounting evidence of operational strain being associated with increased mortality risk [33].

#### The prevalence of variants of concern

Despite the more recent identification of several effective treatments for severe COVID-19, as bed saturation in ICUs again became an issue from November 2020 to January 2021, mortality rates increased across England to rates similar to that seen during the 1st wave [34]. Notably, B117/Alpha was the most prevalent strain of COVID-19 in the UK during November 2020 [35] and is known to have increased transmission and mortality risk [36]. Although we attempted to limit the potential impact of varying prevalence of different variants of concern (VOC), using specific date windows for our analysis and assessing week of admission as a sensitivity analysis, without knowledge of the specific variant COVID-19 for each individual diagnosis, it is impossible to exclude this as a cause of residual confounding.

### Implications for policy makers and clinicians

The results presented in this study are especially relevant given the identification of variants of concern with increased risk of transmission and mortality [36], as well as vaccine/immunity escape potential [37], which suggest that the risk of further epidemics driven by Sars-CoV-2 are not implausible. From a mitigation perspective, our study highlights the importance of public health interventions (such as expeditious vaccination programmes and non-pharmacological interventions), to control both incidence and prevalence of COVID-19, and therefore prevent ICU saturation, as there is evidence of direct harm to patients as a consequence. In terms of adaptation, during the second wave over 1000 patients in critical care units across the UK were transferred due to capacity reasons. Whilst there are pre-existing mechanisms to support these action [38], there is need for more investment in digital infrastructure to support better coordinated action at the national level. Again, the natural experiment produced by the pandemic has produced substantial amounts of data on transferred patients and load-sharing between trusts. The community would benefit greatly from analysis of this real world data to inform future best practices, from whom to consider transferring, how far, as well as a host of other factors that influence decision making, rather than relying solely on model-based inferences which suggest load-sharing is possible and probably effective [39]. Moreover, there are number of other adaptation interventions that might be worth considering for future research, such as modified treatment strategies with lower sedation doses and more aggressive extubation strategies to increase discharge rates during periods of high occupancy and therefore an increased baseline risk of mortality independent of individual-level factors.

## Conclusion

The results of this study suggest that increasing occupancy of beds compatible with mechanical ventilation, a proxy for operational strain, is associated with a higher mortality risk for individuals admitted to ICU. Further research is required to establish if this is a causal relationship or whether it reflects strain on other operational factors such as staff/residual confounding not addressed due to the lack of physiological severity data. If causal, the result highlights the importance of strategies to keep ICU occupancy low to mitigate the impact of this type of resource saturation, rather than waiting for arbitrary thresholds to be triggered, especially given the observation that average exposure (over an individual’s total length of stay) to this operational risk factor is also associated with mortality risk.

## Supplementary Information

**Additional file 1:. eMethods, Figures S1-8**, & **Tables S1-6.** eMethods – Additional detail on data handling and modelling methods. **Fig S1.** - Application of inclusion and exclusion criteria to raw data. **Fig S2.** - Unadjusted mortality rate in the ICU, alongside the total, confirmed COVID and non-COVID national-level occupancies (Top) and percentages of the study cohort in each of the three main ICU occupancy bins used in the primary model (Bottom) across the duration of the study. **Fig S3.** - Trust-level missingness at varying inclusion thresholds. **Fig S4.** - Seven-day rolling averages for Age (Top) and Number of Comorbidities (Bottom) across the duration of the study. **Fig S5.** - Trust-Level Ventilator Bed Occupancy (Based on Surge Capacities) Across England. **Fig S6.** - Marginal posterior densities for linear population level coefficients in the model, for mechanically ventilated bed occupancy binned as described in the main text, comorbidities, sex and week of admission. **Fig S7.** - Marginal posterior densities for group level ethnicity intercepts. **Fig S8.** - The interaction between the baseline availability of beds supporting mechanical ventilation, and occupancy on the day of ICU admission. **Table S1.** - Marginal posterior densities for occupancy under sensitivity analyses. **Table S2.** - Comparison of prior choices via their effect on primary model OR estimates. **Table S3.** - Marginal posterior densities for mortality ORs when occupancy is utilised at each individual’s final outcome date, rather than their date of admission to ICU. **Table S4.** - Marginal posterior densities for mortality ORs when occupancy is taken as the mean or median occupancy achieved during each patient’s stay. **Table S5.** - Marginal posterior densities for mortality ORs when occupancy is defined as a ratio relative to baseline capacity. **Table S6.** - Marginal posterior densities for mortality ORs including baseline bed availability and interaction with occupancy.

## Data Availability

Data, even a de-identified version, cannot be shared publicly as it was collected by Public Health England (PHE) as part of their statutory responsibilities, which allows them to process patient confidential data without explicit patient consent. Given this particular set of circumstances, it was deemed by PHE to not be appropriate to share the data publically as then they would then be unable to fulfil their duty of care to ensure that the data is used in a manner consistent with the statutory remit under which it was collected. Moreover, to retain the fidelity of the data and given that there are numerous unique cases due to low prevalence in certain geographic areas over the time period we analyse, de-identification would be insufficient to comply with the minimum Office for National Statistics’ disclosure principles which would come with significant risk of re-identification. Data utilised in this study were made available through an agreement between the University of Warwick and PHE. Individual requests for access to the raw data are considered directly by PHE (contact via covid19surv@phe.gov.uk).

## Abbreviations

ICU: Intensive care units
CHESS: COVID-19 Hospitalisation in England Surveillance System
SitRep: Daily situation reports
NHS: National Health Service
STPs: Sustainability and transformation partnership
IMD: Indices of multiple deprivation
OR: Odds ratio
PCI: Posterior credibility interval
IQR: Interquartile range
VOC: Variant of concern
ICNARC: Intensive Care National Audit and Research Centre
